# Zinc Deficiency and Zinc Therapy Efficacy with Reduction of Serum Free Copper in Alzheimer's Disease

**DOI:** 10.1155/2013/586365

**Published:** 2013-10-10

**Authors:** George J. Brewer, Sukhvir Kaur

**Affiliations:** ^1^Department of Human Genetics, University of Michigan Medical School, Ann Arbor, MI 48109, USA; ^2^Monmouth University, West Long Branch, NJ 07764, USA

## Abstract

We are in the midst of an epidemic of Alzheimer's disease (AD) in developed countries. We have postulated that ingestion of inorganic copper from drinking water and taking supplement pills and a high fat diet are major causative factors. Ingestion of inorganic copper can directly raise the blood free copper level. Blood free copper has been shown by the Squitti group to be elevated in AD, to correlate with cognition, and to predict cognition loss. Secondly, we have shown that AD patients are zinc deficient compared to age matched controls. Zinc is important in neuronal protection. We carried out a 6-month small double blind trial of a new zinc formulation on AD patients. We found that in patients 70 years and older, zinc therapy protected against cognition decline compared to placebo controls. We also found that zinc therapy significantly lowered blood free copper levels. So zinc efficacy could be due to restoring neuronal zinc levels, to lowering blood free copper levels, or to both.

## 1. Introduction

We are in the midst of an epidemic of Alzheimer's disease (AD), particularly in developed countries [1]. We have previously hypothesized that ingestion of inorganic copper from drinking water and supplement pills and a high fat diet are main causal factors in AD [2–5]. These two factors interact synergistically because copper oxidizes fats to molecules that are toxic, particularly to neurons. The epidemic correlates temporally with the use of copper plumbing in developed countries, and there is a great amount of additional data that draws the net tighter around a causative role for inorganic copper. Inorganic copper must not be confused with organic copper, the copper in food that is safely bound to protein. Inorganic copper is a simple salt of copper, not bound to anything, and is in part handled differently by the intestinal absorption mechanism, such that some of it contributes immediately to the serum free copper pool [6]. This pool has been shown by Squitti and her group to be expanded in AD [7], to correlate negatively with cognition [8], and to predict deterioration in cognition [9]. So, our inorganic copper hypothesis fits well with the work of the Squitti et al. group.

 While we believe ingestion of inorganic copper and a high fat diet are major causal factors, there are a number of other known risk factors in AD. These include having an apolipoprotein E4 allele [10], having elevated homocysteine levels [11], or having certain alleles of the iron management genes, hemochromatosis [12] and transferrin [13]. These latter fit with the copper hypothesis since iron and copper are toxic through the same mechanism, generation of oxidant damage. In addition, the Squitti group has shown that possession of certain variants of the ATP7B gene is a risk factor for AD [14]. Since ATP7B gene products control copper levels in the body, this risk factor also fits beautifully with the copper hypothesis.

 A risk factor for AD that we have recently observed has largely been ignored by the scientific community concerned with AD. This is zinc deficiency in AD patients. We have found [15], as has another group [16], that AD patients are zinc deficient. Zinc has many protective roles in neurons, and zinc deficiency may play a causal role in AD. We have found and will report here that zinc therapy appears to prevent at least some cognition decline. Our findings on zinc therapy may also lend further support to the copper hypothesis, because we have found that zinc therapy, in addition to restoring normal zinc levels, reduces serum free copper levels in AD.

## 2. Demonstration of Zinc Deficiency in AD 

The following are the highlights of our study [15]. A total of 29 patients with AD, diagnosed by standard criteria, were compared to 29 age and sex matched controls. Since elderly people tend to take a variety of supplements, many of which contain copper or zinc, we halted all supplement use beginning 30 days prior to the study, the first time that has been done in studying AD patients, to our knowledge. Serum zinc was assayed by atomic absorption.

Serum zinc declines with age in people for unknown reasons. Thus, in young adults, the mean serum zinc is around 100 *μ*g/dL. In our group of 29 normal but aged controls, the mean was well below that at 82.7 *μ*g/dL. But the mean of the 29 AD patients, 76.2 *μ*g/dL, was significantly (*P* = 0.027) below the mean of the normal aged group. Thus, the AD patients are relatively zinc deficient compared to age matched controls. It should be kept in mind that serum zinc is a relatively late indication of zinc deficiency, but when it is low, the patient is zinc deficient.

## 3. Is Adequate Available Zinc Important in Neuronal Health?

The neurons of many parts of the brain have high zinc levels, and it is clear that zinc plays many critical roles in neurons. In some neurons, high concentrations of zinc are secreted along with glutamate into the synapse. Glutamate initiates firing, and zinc quenches, or shuts down, the firing [17]. With inadequate zinc, glutamate-induced firing persists and can damage the neuron. Excess glutamatergic excitotoxicity is believed to be a common occurrence in many neurodegenerative disorders, including AD. 

Another possible mechanism by which low availability of zinc in the brain can have harmful effects is through failure of adequate inhibition of calcineurin [18]. Calcineurin activity is known to be high in AD brain, and this can have many negative downstream effects. Calcineurin is normally inhibited by zinc but is stimulated by beta amyloid.

Additional information about the key role of zinc in the brain is provided by the studies of Adlard et al. [19] in which they did a knockout of mice of the zinc transporter-3 (ZnT3) gene. ZnT3 is the pump that puts zinc into the vesicle to be discharged into the synapse. These mice, with a deficiency of zinc in the synapse, are a phenocopy of deficits in AD. 

To add to the problem created by systemic zinc deficiency, there is another mechanism in the AD brain that depletes neurons of much-needed zinc. The beta amyloid plaques, that build up in the AD brain, are avid binders of zinc [19]. Thus, it seems very likely that the neurons of the AD brain are seriously lacking in available zinc and probably many are injured and die as a result.

## 4. Random Controlled Trial of Zinc Therapy 

Given the above discussion of the critical roles of zinc in maintaining healthy neurons and the demonstration that AD patients are zinc deficient, it is logical to consider a trial of zinc therapy in AD. Interestingly, an uncontrolled trial of zinc therapy, both oral and parenteral, was carried out in 1992, with reportedly dramatic positive effects [20]. The uncontrolled nature of this study detracts from its significances, but it is puzzling that this very positive study has not been followed up. The probable reasons are that controlled drug trials are expensive and that the lack of a patent on zinc makes it unattractive to drug companies. In addition to the former human work, there has been a positive animal study, in which zinc supplementation caused improvement in an AD animal model [21].

We decided to pursue a controlled zinc trial in AD. By way of background, one of us (G. J. Brewer) had developed zinc therapy for Wilson's disease, an inherited disease of copper accumulation and copper toxicity [22]. Zinc acts in this disease by blocking intestinal copper absorption. We developed a zinc acetate salt, with the trademark name, Galzin, that was approved by the FDA for Wilson's disease in 1997. While effective, Galzin had to be given three times a day in 50 mg doses and each dose had to be separated from food in order to have the desired effect in the intestine. It turned out that up to 50% of patients had some gastric discomfort from Galzin and up to 10% could not take it at all. The problem was that the zinc capsules dissolved in one spot in the empty stomach, releasing all the zinc salt in that location, causing irritation in many patients. 

At Adeona Pharmaceuticals, we developed a new zinc formulation with a zinc-binding agent that allowed the slow release of zinc. The kinetics of this formulation are such that in dissolution studies, peak zinc release is at 6–8 hours, and it is still releasing zinc at 24 hours. Thus, this pill caused no gastric intolerance, and because of the slow release, could be given one time/day, with around-the-clock elevation of serum zinc. We used this new zinc formulation in the controlled AD trial reported here.

### 4.1. Methods

The study was carried out in Clearwater, Florida, USA with Dr. Diana Pollock, of the Ptak Alzheimer's Center, Morton Plant Neuroscience Institutes, Morton Plant Hospital, Clearwater, FL, USA as the principal investigator. It was designed to recruit 60 AD patients. Patients were diagnosed using Alzheimer's Disease and Related Disorders Association criteria. They had mild to moderate AD with a Clinical Dementia Rating (CDR) of 0.5–1.5 and a mean Mini Mental State Examination (MMSE) of 25.6. Patients were randomly assigned to receive either 150 mg of the new zinc formulation in a once-a-day dose or matching placebo. The end points were improvement in cognition relative to controls, improvement in serum zinc, and reduction in serum free copper. Cognition was measured by three tests, the Alzheimer's Disease Assessment Scale (ADAS-Cog), the Mini Mental State Examination (MMSE), and CDR Sum of Boxes (CDR-SOB). Treatment was for 6 months. Zinc and copper were assayed by atomic absorption spectroscopy. Ceruloplasmin was measured by a nephelometric immunologic technique [23]. Student's *t*-test was used to test for statistical significance which was set at *P* at or below 0.05. 

### 4.2. Results

Since this is a high dose of zinc, patients were monitored for copper deficiency by following serum ceruloplasmin. Either serum ceruloplasmin or serum copper can be followed for this purpose since almost all serum copper is in ceruloplasmin. We choose ceruloplasmin because the assay is more generally available in clinical laboratories. Only one patient had a sufficient decrease in ceruloplasmin to merit a reduction in zinc dose to 75 mg/day. This patient's data were included in the results of the study. The data on cognition were tested for statistical significance by Student's *t*-test. As reported by Dr. Pollock at the 2011 meeting of the American Academy of Neurology in Hawaii, there was a trend in the data from all three cognition screening tests favoring the zinc-treated group versus the placebo group. The *P* values for ADAS-Cog (*P* = 0.36), MMSE (*P* = 0.42), and CDR-SOB (*P* = 0.1) were not statistically significant although CDR-SOB was close at *P* = 0.1. 

In examining the data, we noted that zinc was essentially stabilizing the cognition in patients, both young and old. However, in the placebo group, younger patients did not show much cognition change, but at about age 70 and older, they showed considerable deterioration. So, we analyzed the data comparing the two groups looking at patients aged 70 and over, the concept being that the greater variability and inconsistency in deterioration in the younger placebo patients were preventing the differences from being significant.

The very exciting results are shown in Table 1. With ADAS-Cog, a more positive change over the 6-month period indicates deterioration, and the placebo group deteriorated +1.27 points while the treatment group actually improved −0.76 points with a statistically significant difference at *P* = 0.037. With CDR-SOB, a more positive change again indicates deterioration, and the placebo group deteriorated +0.87 points, while the treatment group deteriorated significantly less (*P* = 0.032). With MMSE, a negative change indicates deterioration and the placebo group deteriorated −1.0 points while the treatment group actually improved to +0.58 points, but results were not quite statistically significant at *P* = 0.067.

We also reached our end points with serum zinc and serum free copper. In the placebo group, baseline serum zinc was 70.8 *μ*g/dL, and 6 months later, it was 75.3 (*P* = 0.153). In the zinc treatment group, baseline serum zinc was 76.4, and 6 months later, it was 151.8 (*P* = 0.002). Regarding serum free copper in the placebo group, baseline serum free copper was 34.8 *μ*g/dL, and 6 months later, it was 34.9 (*P* = 0.486). In the zinc-treated group, baseline serum free copper was 37.0, and 6 months later, it was 30.8 (*P* = 0.004). So, zinc deficiency was very effectively eliminated and serum free copper was very significantly lowered by zinc therapy.

## 5. Discussion 

The first question to discuss is whether we now know for sure that zinc therapy will slow or halt cognitive decline in AD. At this point, we cannot give an absolute “yes”; the answer has to be qualified. We think the data are quite strong that this is the case. First, there is the randomized controlled trial (RCT) just reviewed in the previous sections. Second, there are the 1992 uncontrolled studies of Constantinidis [20], which were strongly positive. Third, there are the animal model studies of Corona et al. [21], in which zinc supplementation significantly improved cognition. Fourth, there are the ZnT3 mouse knockout studies of Adlard et al. [19], in which mice deprived of much of their neuronal zinc were a phenocopy of AD. Fifth, there are clear data that AD patients are more zinc deficient than age matched controls [15, 16]. Sixth, there are all the known protective roles of adequate zinc levels in the neuron, which were reviewed earlier.

So, the evidence is quite strong, with the RCT being the potential linchpin for a “yes” answer to the question. But the weakness of the RCT is that the statistically significant benefit to cognition was a post hoc analysis of the data on those aged 70 and over. Why are post hoc data weaker than pre hoc hypothesis testing? The major factor is that if you manipulate data in multiple ways, it becomes increasingly likely that one of the analyses will become significant by chance. Our post hoc analysis was limited to two evaluations (the other analysis asked whether there was a difference in results between the two sites of the study). So the *P* value is not weakened very much.

The other factor to consider about our RCT is that the end sample of patients is rather small, 14 AD patients of age 70 or over who were treated with zinc, and 15 AD patients of age 70 or over who were controls.

A factor which strengthens the conclusion of efficacy of zinc is the consistency of the three cognitive scoring tests. All three indicated benefit to the zinc-treated group. Two were strongly statistically significant, and one was close. If chance were the main operative, one would suspect heterogeneity among the tests. The consistency strongly supports that a biologic signal was operating, that is, a positive effect of zinc therapy on protecting cognition.

So, at this point we draw the conclusion, admittedly not quite final yet, that zinc therapy significantly slows cognition loss in AD.

The second question to discuss, assuming zinc efficacy, is what is the mechanism? In the first part of this paper, we laid out the background for two likely mechanisms. First, we laid out our hypothesis that ingestion of inorganic copper, such as from drinking water or taking supplement pills, increases the serum free copper level. Squitti and her group have shown in several elegant studies that serum free copper is high in AD [7], that it is correlated with cognition measures [8], and it is predictive of cognition loss over time [9]. These data indicate that elevated serum free copper is causative of cognition decline in AD. We significantly lowered serum free copper in the zinc-treated group; thus, a mechanism of benefit from zinc is very likely reduction in serum free copper.

However, earlier in the paper, we also pointed out that AD patients are more zinc deficient than age matched controls, and that zinc deficiency by amyloid plaques further depletes the neurons of zinc, and we pointed out how important adequate zinc is for neuronal health. Then there is the ZnT3 knockout mice that produce a phenocopy of AD [19]. The neurons in AD patients are very likely zinc deficient, so a case can be made that restoring zinc to the neurons of AD patients was a mechanism for improved cognition in the zinc group in our trial.

Or perhaps it is both the beneficial lowering of serum free copper and the beneficial restoration of neuronal zinc that played a role in the zinc therapy benefit we saw. This can be studied by a trial in which we have three groups of patients, a control group, a group receiving zinc as in the trial described, and a group getting zinc as in group two plus a copper supplement to maintain baseline serum free copper.

Such a trial would be expensive. Perhaps of higher priority is a trial to simply confirm zinc benefit in a larger number of patients over a longer period of time. We are not aware of any plans for such a trial at present. In the meantime, our study is there for all to see.

## Figures and Tables

**Table 1:** Post hoc analysis of cognition change in patients 70 years and older during the six-month controlled trial.

Cognition test	Direction of deterioration	Zinc-treated group (*N* = 14)	Placebo group (*N* = 15)	*P* value
ADAS-Cog	Positive change	−0.76	+1.27	0.037
CDR-SOB	Positive change	+0.25	+0.87	0.032
MMSE	Negative change	+0.58	−1.0	0.067
